# Effectiveness of tailored screening for multidrug-resistant organisms upon admission to an intensive care unit in the United Arab Emirates

**DOI:** 10.1186/s13756-023-01340-x

**Published:** 2023-11-28

**Authors:** Sungsoo Park, Heesuk Kim, Katrine K. Gatchalian, Hyeyoung Oh

**Affiliations:** 1 Division of Medicine, Sheikh Khalifa Specialty Hospital, Al Shohadaa Road, PO Box 6365, Ras Al Khaimah, UAE; 2 Department of Pulmonology, Seoul National University Hospital, Seoul, Republic of Korea; 3 Environmental Safety Healthcare Provider Team, Sheikh Khalifa Specialty Hospital, Ras Al Khaimah, UAE; 4 Department of Family Medicine, Seoul National University Hospital, Seoul, Republic of Korea

**Keywords:** Antimicrobial drug resistance, Screening, Multidrug-resistant organism, Intensive care units

## Abstract

**Background:**

Multidrug-resistant organism (MDRO) screening may identify high-risk patients for MDRO infection and curb the spread of these resistant pathogens. However, the heterogeneous practices in MDRO screening and the diversity of MDRO risk factors necessitate a tailored approach for successful implementation. This study aimed to evaluate the performance of tailored MDRO screening in predicting MDRO carriage compared to universal screening.

**Methods:**

Critically ill patients who underwent MDRO screening tests upon intensive care unit admission between September 2015 and December 2019 were included in the study. A risk-predicting model was developed using risk factors identified through multivariable logistic regression analysis. If an individual had one or more identified risk factors, the individual was deemed to be at risk of MDRO carriage and undergo tailored screening. The sensitivity of tailored screening was compared with universal screening for methicillin-resistant *Staphylococcus aureus* (MRSA) and multidrug-resistant Gram-negative bacilli (carbapenem-resistant *Acinetobacter baumannii* and carbapenem-resistant Enterobacterales).

**Results:**

The use of tracheostomy or endotracheal tubes, previous antibiotic exposure, previous multidrug-resistant Gram-negative bacilli carriage history, admission to the medical department, peripheral vascular disease, and liver disease were associated with positive screening for multidrug-resistant Gram-negative bacilli. These six risk factors accounted for all positive screening for multidrug-resistant Gram-negative bacilli, requiring 38.6% of all tests. Notably, MRSA had different risk factor profiles, and the risk factor-based screening approach detected only 43.1% (31 out of 72) of MRSA-positive cases.

**Conclusions:**

Tailored screening based on identified risk factors showed variable sensitivities to individual MDROs compared to universal screening. A tailored screening approach for individual MDROs may enhance the overall effectiveness of MDRO screening programs.

**Supplementary Information:**

The online version contains supplementary material available at 10.1186/s13756-023-01340-x.

## Background

Antimicrobial resistance (AMR) has emerged as a global public health threat, which has increased more during the coronavirus 2019 pandemic [1]. Bacterial AMR poses significant challenges, including prolonged hospital stays, increased healthcare costs, and higher mortality rates [2–4]. To address this issue, the World Health Organization has launched a global action plan to tackle infections by multidrug-resistant organisms (MDROs) [5]. This comprehensive plan encompasses various strategies, including raising awareness about AMR, strengthening knowledge through surveillance, reducing infection incidence, optimizing antimicrobial usage, and developing the economic case for sustainable investment.

Early identification of high-risk patients for MDRO infection is crucial to curb the spread of MDROs and minimize their detrimental impact on patient outcomes [6, 7]. Several practice guidelines have suggested systemic MDRO screening of at-risk patients as one of the vital preventive measures to detect MDRO carriers [8, 9]. However, the effectiveness and benefits of this screening strategy have yet to be thoroughly validated. Previous studies assessing the screening strategy were conducted under diverse epidemiological situations and employed varying criteria for selecting cases and controls, making it difficult to draw a solid conclusion [10]. Additionally, the screening approach requires high costs, increased workload for microbiological staff, and diversion of resources from other essential services.

The Gulf Cooperation Council (GCC), a regional organization in the Middle East comprising Saudi Arabia, the United Arab Emirates, Kuwait, Qatar, Bahrain, and Oman, have documented a considerable burden of AMR in 2016 [11, 12]. The GCC prompted the GCC Center for Infection Control to announce a strategic plan for AMR. One of the key suggestions was adopting a unified method to conduct systemic MDRO surveillance. However, there may be heterogeneous practices in MDRO screening and diverse risk factors for MDRO colonization in the Gulf region [13]. Consequently, a tailored approach based on the prevalence of MDROs, patient-level risk factors, and hospital-specific characteristics is required to implement the MDRO screening program successfully [14].

Sheikh Khalifa Specialty Hospital is a 246–bed tertiary care hospital in the Northern Emirates with a total of 28 intensive care unit (ICU) beds, comprising 10 coronary care unit beds and 18 surgical and medical ICU beds. The hospital has implemented universal MDRO screening tests upon admission to the ICU since September 2015. This screening protocol consists of 4 items to detect methicillin-resistant *Staphylococcus aureus* (MRSA), carbapenem-resistant Enterobacterales (CRE), carbapenem-resistant *Acinetobacter baumannii* (CRAB), and vancomycin-resistant Enterococcus (VRE). However, the observed MDRO carriage rate has been low, except among bedridden patients with frequent antibiotic use and multiple hospitalizations. As a result, the hypothesis was proposed that tailored screening based on MDRO risk factors would yield comparable results to universal screening in detecting MDRO colonization. This study aimed to assess the performance of tailored screening in detecting MDRO carriage relative to universal MDRO screening.

## Materials and methods

### Study population and data collection

**Fig. 1:**
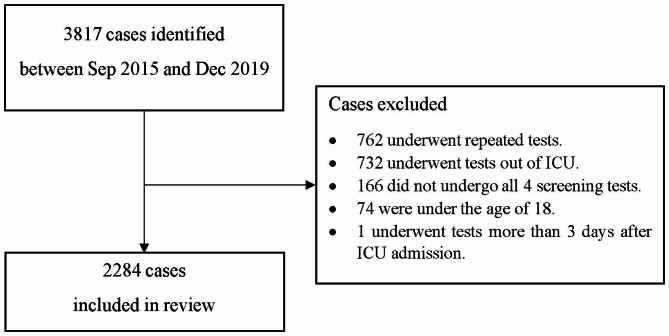
Flow diagram for case identification

This retrospective study included all critically ill patients who underwent 4 MDRO screening tests upon ICU admission between September 2015 and December 2019 (Fig. 1). Subjects who did not complete four required tests, were under 18, and took tests more than three days after ICU admission were excluded. If a patient was admitted to ICU more than once, only initial tests were included in the analysis.

Demographic information and MDRO risk factors were obtained from the electronic medical records. Demographic data included age, sex, comorbidities, Acute Physiology and Chronic Health Evaluation (APACHE) II score, reason for ICU admission, and admitting department. The MDRO risk factors collected were previous antimicrobial therapy within the past three months, previous hospital, healthcare facility, and ICU admission or surgery within the past year, and prior placement of medical devices, such as central lines, hemodialysis catheters, urinary catheters, tracheostomy tubes, and gastrostomy tubes) within the last year, and previous MDRO carriage. Previous MDRO carriage history only included a history of MRSA, CRE, CRAB, and VRE. Since many patients stayed in another hospital for 1–2 days and were transferred to our institution with newly inserted catheters, patients who stayed in a hospital or had medical devices for at least 3 days were deemed to have an admission history and placement of medical devices.

### MDRO testing & reporting

Our institution implemented MDRO screening for all patients upon ICU admission on 17 September 2015. The screening process involved collecting nasal swabs for MRSA, rectal swabs for CRE, throat swabs for CRAB, and rectal swabs for VRE. These swabs were inoculated onto CHROMagar MRSA, CRE, ACINETO, and VRE (CHROMagar Microbiology, Paris, France) and incubated in aerobic conditions at 35 ± 2 °C for 18 to 48 h. The plates were then examined for the amount of growth and color formation. To confirm the results, identification and antibiotic sensitivity testing were performed using VITEK®2 cards (bioMérieux, Marcy-l’Etoile, France).

### Statistical analysis

The chi-square (𝜒^2^) or Fisher’s exact test, as appropriate, and Mann–Whitey tests were used to compare categorical and continuous data between the MDRO-positive group (positive group thereafter) and the MDRO-negative group (negative group thereafter), respectively. Since MRSA and multidrug-resistant Gram-negative bacilli (CRE and CRAB) have different epidemiologic and microbiologic characteristics, modelling was conducted for MRSA and multidrug-resistant Gram-negative bacilli separately. VRE was excluded from the analysis as few patients had VRE.

The associations between positive MDRO screening and relevant variables were assessed using multivariable logistic regression. Variables with P values ≤ 0.1 in the univariate analysis were entered in a forward stepwise logistic regression analysis. A p-value of ≥ 0.10 was selected as the parameter exclusion criterion in the forward selection, generating a prediction model of MDRO carriage. Possible effect modification was assessed by fitting interaction terms between variables and comparing resulting models by likelihood ratio tests.

If an individual had one or more identified risk factors, the individual was deemed to be at risk of MDRO carriage and undergo tailored screening. To evaluate the performance of tailored screening, we compared its sensitivity and the number of patients required to be screened with those of the universal screening model.

A two-sided alpha level of 0.05 defined statistical significance. The analyses were conducted using R statistical software (RStudio, version 0.98.1103, Boston, MA).

## Results

### Characteristics of the study population

Of the 2284 cases who underwent MDRO screening, the median age was 59.0 years (interquartile range, IQR 45.0–72.0), and 650 (28.5%) were females (Table 1). Positive cases for MRSA, CRE, CRAB, and VRE screening on ICU admission were observed in 72 (3.2%), 39 (1.7%), 15 (0.7%), and 4 (0.2%) patients, respectively.

**Table 1:** Characteristics of the patients at baseline

	Total (N = 2284)	Tested positive* (N = 128)	Tested negative (N = 2156)	P value
**Age, median (IQR), years**	59.0 (45.0, 72.0)	66.5 (55.0, 76.8)	58.0 (45.0, 71.0)	< 0.001
**Female**	650 (28.5%)	42 (32.8%)	608 (28.2%)	0.261
**Reason for admission**				< 0.001
Postoperative monitoring	1341 (58.7%)	45 (35.2%)	1296 (60.1%)	
Acute respiratory failure	386 (16.9%)	43 (33.6%)	343 (15.9%)	
Sepsis/Septic shock	152 (6.7%)	17 (13.3%)	135 (6.3%)	
Circulatory failure	96 (4.2%)	9 (7.0%)	87 (4.0%)	
Neurogenic failure	59 (2.6%)	5 (3.9%)	54 (2.5%)	
**Admitted department**				< 0.001
Cardiology	1256 (55.0%)	33 (25.8%)	1223 (56.7%)	
Surgical department	306 (13.4%)	16 (12.5%)	290 (13.5%)	
Medical department	722 (31.6%)	79 (61.7%)	643 (29.8%)	
**APACHE II score (N = 2082)**	9 (6, 15)	16 (9, 23)	9 (6, 15)	< 0.001
**MDRO risk factors**				
Admission history	772 (33.8%)	85 (66.4%)	687 (31.9%)	< 0.001
Surgical history	201 (8.8%)	25 (19.5%)	176 (8.2%)	< 0.001
Use of any catheter	365 (16.0%)	64 (50.0%)	301 (14.0%)	< 0.001
Previous antibiotic exposure	486 (21.3%)	71 (55.5%)	415 (19.2%)	< 0.001
Previous MDRO carriage history	65 (2.8%)	26 (20.3%)	39 (1.8%)	< 0.001
**Comorbidities**				
Diabetes mellitus	991 (43.4%)	59 (46.1%)	932 (43.2%)	0.525
Myocardial infarction	435 (19.0%)	22 (17.2%)	413 (19.2%)	0.582
Chronic kidney disease	168 (7.4%)	12 (9.4%)	156 (7.2%)	0.368
Stroke	169 (7.4%)	26 (20.3%)	143 (6.6%)	< 0.001
Peripheral vascular disease	56 (2.5%)	11 (8.6%)	45 (2.1%)	< 0.001
Liver disease	38 (1.7%)	9 (7.0%)	29 (1.3%)	< 0.001
Cancer	227 (9.9%)	19 (14.8%)	208 (9.6%)	0.056

*Note*: IQR = interquartile range, APACHE = acute physiology and chronic health evaluation, MDRO = multidrug-resistant organisms

* Positive for any MDROs (MRSA, CRE, CRAB, and VRE)

The most common cause of ICU admission was postprocedural or postoperative monitoring, accounting for 1341 cases (58.7%), followed by acute respiratory failure with 386 cases (16.9%). Patients who were admitted to cardiology, surgical departments, and medical departments were 1256 (55.0%), 306 (13.4%), and 722 (31.6%), respectively. Approximately one-third of patients had a history of admission, and 8.8% underwent surgery within the past year. 16% had at least one catheter, and 21% received antibiotics within the last 3 months. Only 2.8% had any of the 4 MDROs previously.

### Risk factors related to positive MDRO screening

The positive group had a higher median age and APACHE II scores than the negative group (Table 1). The positive group was more likely to be admitted to medical departments with acute respiratory failure and sepsis from general wards. Five MDRO risk factors were more prevalent in the positive group, including admission history, surgical history, use of any catheter, previous antibiotic exposure, and previous MDRO carriage history.

The CRE and CRAB-positive groups demonstrated similar characteristics to the MDRO-positive group (Additional file 1: Table S1). In contrast, the MRSA-positive group predominantly consisted of patients admitted to the cardiology department for postoperative monitoring. These MRSA-positive patients had fewer prior admissions, less frequent catheter usage, and a lower history of previous antibiotic exposure than the CRE and CRAB-positive groups.

### Performance of a risk-prediction model

The use of tracheostomy or endotracheal tubes, previous antibiotic exposure, previous CRE or CRAB carriage history, and admission to the medical department were related to positive CRE or CRAB screening, with adjusted odds ratios of 20.017 (95% CI: 8.003–50.065, *p* < 0.001), 3.691 (95% CI: 1.413–9.640, *p* = 0.008), 13.329 (95% CI: 5.597–31.741, *p* < 0.001), and 6.601 (95% CI: 1.366–31.907, *p* = 0.019), respectively (Table 2). The adjusted odds ratios of PVD and liver disease were also 4.704 (95% CI: 1.433–15.444, *p* = 0.011) and 3.933 (95% CI: 1.078–14.358, *p* = 0.038), with association with CRE or CRAB carriage. Admission history, surgical history, and stroke did not show significant association with CRE or CRAB carriage.

On the contrary, when repeating the analysis on MRSA, prior MRSA carriage history and liver disease were only associated with positive MRSA screening (adjusted odds ratio of 12.262; 95% CI: 4.982–30.184, *p* < 0.001 and 4.247; 95% CI: 1.455–12.395, *p* = 0.008, respectively).

**Table 2:** Adjusted odds ratios for positive MDRO screening

	Unadjusted OR (95% CI)	P value	Adjusted OR (95% CI)	P value
**CRE or CRAB**				
Use of tracheostomy or endotracheal tube	30.232 (16.694, 54.747)	< 0.001	20.017 (8.003, 50.065)	< 0.001
Previous antibiotic exposure	19.788 (9.588, 40.838)	< 0.001	3.691 (1.413, 9.640)	0.008
Previous CRE or CRAB carriage history	45.333 (22.330, 92.034)	< 0.001	13.329 (5.597, 31.741)	< 0.001
Admission to the medical department	40.690 (9.834, 168.362)	< 0.001	6.601 (1.366, 31.907)	0.019
Peripheral vascular disease	6.776 (2.914, 15.761)	< 0.001	4.704 (1.433, 15.444)	0.011
Liver disease	6.938 (2.596, 18.544)	< 0.001	3.933 (1.078, 14.358)	0.038
**MRSA**				
Previous MRSA carriage history	10.720 (4.422, 25.990)	< 0.001	12.262 (4.982, 30.184)	< 0.001
Liver disease	3.768 (1.301, 10.917)	0.015	4.247 (1.455, 12.395)	0.008

*Note*: MDRO = multidrug-resistant microorganism, OR = odds ratio, CI = confidence interval, CRE = carbapenem-resistant Enterobacterales, CRAB = carbapenem-resistant *Acinetobacter baumannii*, MRSA = methicillin-resistant *Staphylococcus aureus*

Tailored screening utilizing the six CRE or CRAB risk factors detected all cases (53/53) with positive multidrug-resistant Gram-negative bacilli, requiring 882 tests, constituting 38.6% of all tests. However, for MRSA, the tailored screening approach detected only 43.1% (31/72) of MRSA-positive cases.

## Discussion

Our study investigated the association between MDRO screening results and various risk factors upon ICU admission and the performance of a tailored screening strategy for the 4 MDROs. Previous studies have highlighted that recent antibiotic exposure, previous admission, age, and sex were the most common risk factors for MDRO carriage [10]. Our study confirmed the role of previous antibiotic exposure as an MDRO risk factor, while the other mentioned risk factors were insignificant. This discrepancy may reflect the diverse nature of MDRO risk factors, which can vary based on the region and population.

We observed a positive association between PVD and liver disease with positive MDRO screening. These comorbidities may be related to frequent contact with healthcare facilities and antibiotic use, leading to MDRO carriage. PVD and liver disease may also serve as proxies for underlying conditions such as foot ulcers, wounds, and immunosuppression, which can influence MDRO carriage [15]. Recent studies have shown an increased risk of colonization during hospitalization among patients with PVD [16] and a high prevalence of MDRO carriage among patients with cirrhosis [17, 18]. However, due to the small number of PVD and liver disease cases in our cohort, caution is needed when interpreting these findings.

Interestingly, 57% of patients with MRSA had no known classical MDRO risk factors, suggesting the presence of community-associated MRSA. This aligns with a recent study indicating the emergence of community-associated MRSA with a 25–35% prevalence in the Gulf Cooperation Council countries [19]. The risk factors related to the community strains are injection drug use, exposure to correctional facilities, crowding, and unstable housing [14]. However, these risk factors were not evident in our cohort, which poses challenges in identifying the population to be screened. Community strains may be imported from patients’ home countries or acquired during travel [12].

The effectiveness of tailored screening for MRSA, based on identified risk factors, was notably reduced in our study, possibly due to community-associated MRSA, resulting in a moderate sensitivity for detecting any MDRO. The lower sensitivity of clinical prediction tools (30-81.3%) for identifying MRSA carriers in a recent meta-analysis may be partially explained by community-associated MRSA [10]. Therefore, a combination of universal screening for MRSA and tailored screening for CRE could detect most cases without significantly increasing the number of patients screened. This strategy may be particularly beneficial in regions with a high incidence of community-associated MRSA and relatively low prevalence of other MDROs. In addition, cost savings can be achieved by conducting only 38.6% of tests to detect all CRE-positive cases. However, the cost-effectiveness of each MDRO and its impact on patient outcomes should be taken into account, as some MDRO colonization was not associated with adverse outcomes in certain situations [20].

The strength of our study lies in the inclusion of a large cohort of critically ill patients enrolled over five years. However, it is difficult to generalize the findings in a single-center retrospective study. The cohort in our study had a female representation of less than 30%. This gender distribution could be attributed to the substantial predominance of males (80.2%) among patients admitted to cardiology department and the high male-to-female ratio in the United Arab Emirates. Our study did not assess other MDROs, such as extended-spectrum beta-lactamase-producing bacteria and multidrug-resistant *Pseudomonas aeruginosa*. Obtaining more detailed information on admission, surgery dates, placed catheters, and antibiotics from other hospitals was challenging. Nonetheless, the consistency of our findings with previous studies strengthens the reliability of the results.

## Conclusions

Tailored MDRO screening based on identified risk factors showed variable sensitivities to individual MDROs compared to universal screening. The variable sensitivities suggested that a tailored approach to each MDRO may enhance the effectiveness of MDRO screening. Implementing such an approach could lead to improved effectiveness of active surveillance and more efficient resource utilization. Further prospective research is needed to validate these findings in diverse healthcare settings and explore the impact of tailored screening on patient outcomes and healthcare practices.

### Electronic supplementary material

Below is the link to the electronic supplementary material.

Supplementary Material 1: Baseline characteristics of patients by each MDRO.

## Data Availability

The datasets used and/or analyzed during the current study are available from the corresponding author upon reasonable request.
