# Supplementary material for: Effectiveness of tailored screening for multidrug-resistant organisms upon admission to an intensive care unit in the United Arab Emirates
Source: Antimicrob Resist Infect Control. 2023 Nov 28;12:135. doi: 10.1186/s13756-023-01340-x (PMC10683294; doi:10.1186/s13756-023-01340-x)
Supplement: Supplementary file 1 — Supplementary Material 1: Baseline characteristics of patients by each MDRO. [file 13756_2023_1340_MOESM1_ESM.docx]

**Supplemental Table 1** Baseline characteristics of patients by each MDRO

|  | | **MRSA**  **(N=72)** | **CRE**  **(N=39)** | **CRAB**  **(N=15)** | **VRE**  **(N=4)** |
| --- | --- | --- | --- | --- | --- |
| **Age, median (IQR), years** | | 62.0  (54.0, 73.0) | 75.0  (57.0, 82.0) | 63.0  (56.0, 79.0) | 73.0  (44.5, 76.8) |
| **Female** | | 27 (37.5%) | 11 (28.2%) | 5 (33.3%) | 1 (25.0%) |
| **Reason for admission** | |  |  |  |  |
| Postoperative monitoring | 37 (51.4%) | 6 (15.4%) | 2 (13.3%) | 0 (0%) |  |
| Acute respiratory failure | 19 (26.4%) | 16 (41.0%) | 7 (46.7%) | 1 (25.0%) |  |
| Sepsis/Septic shock | 4 (5.6%) | 10 (25.6%) | 3 (20.0%) | 1 (25.0%) |  |
| Circulatory failure | 6 (8.3%) | 1 (2.6%) | 2 (13.3%) | 0 (0%) |  |
| Neurogenic failure | 1 (1.4%) | 4 (10.3%) | 0 (0%) | 1 (25%) |  |
| **Admitted department** | |  |  |  |  |
| Cardiology | | 31 (43.1%) | 2 (5.1%) | 0 (0%) | 1 (25%) |
| Surgical department | 9 (12.5%) | 6 (15.4%) | 1 (6.7%) | 0 (0%) |  |
| Medical department | 32 (44.4%) | 31 (79.5%) | 14 (93.3%) | 3 (75%) |  |
| **APACHE II score,**  **median (IQR) (N=2082)** | | 13  (8, 20) | 18  (13, 25) | 21  (17, 27) | 16.5  (8.5, 27.5) |
| **MDRO risk factors** | |  |  |  |  |
| Admission history | | 35 (48.6%) | 34 (87.2%) | 14 (93.3%) | 4 (100%) |
| Surgical history | | 12 (16.7%) | 8 (20.5%) | 2 (13.3%) | 3 (75.0%) |
| Use of any catheter | | 20 (27.8%) | 28 (71.8%) | 14 (93.3%) | 3 (75.0%) |
| Previous antibiotic exposure | | 24 (33.3%) | 31 (79.5%) | 14 (93.3%) | 4 (100%) |
| Previous MDRO carriage history* | | 7 (9.7%) | 9 (23.1%) | 8 (53.3%) | 0 (0%) |
| **Comorbidities** | |  |  |  |  |
| Diabetes mellitus | | 34 (47.2%) | 19 (48.7%) | 6 (40.0%) | 2 (50.0%) |
| Myocardial infarction | | 15 (20.8%) | 6 (15.4%) | 1 (6.7%) | 1 (25.0%) |
| Chronic kidney disease | | 4 (5.6%) | 7 (17.9%) | 2 (13.3%) | 1 (25.0%) |
| Stroke | | 8 (11.1%) | 14 (35.9%) | 3 (20.0%) | 2 (50.0%) |
| Peripheral vascular disease | | 4 (5.6%) | 6 (15.4%) | 1 (6.7%) | 0 (0%) |
| Liver disease | | 4 (5.6%) | 3 (7.7%) | 2 (13.3%) | 0 (0%) |
| Cancer | | 12 (16.7%) | 4 (10.3%) | 2 (13.3%) | 1 (25.0%) |

*Note*: MDRO = multidrug-resistant microorganism, MRSA = methicillin-resistant *Staphylococcus aureus*, CRE = carbapenem-resistant Enterobacterales, CRAB = carbapenem-resistant *Acinetobacter baumannii*, VRE = vancomycin-resistant Enterococcus, IQR = interquartile range, APACHE = acute physiology and chronic health evaluation

*MDRO indicates MRSA, CRE, CRAB, and VRE for each MDRO group in order.
